# Supplementary material for: The Role of Speckle Tracking Echocardiography in the Evaluation of Common Inherited Cardiomyopathies in Children and Adolescents: A Systematic Review
Source: Diagnostics (Basel). 2021 Apr 1;11(4):635. doi: 10.3390/diagnostics11040635 (PMC8066718; doi:10.3390/diagnostics11040635)
Supplement: Supplementary file 1 [file diagnostics-11-00635-s001.pdf]

Supplementary Materials

SEARCH STRATEGY

Search strategy – PROSPERO protocol upload – 21.02.2020

Search strategy modified on 08.05.2020 – for PubMed and EMBASE some human studies were not indexed correctly, search repeated for PubMed and EMBASE inception->02.2020 for studies NOT human, and entries added to screening (n= 51 and 46 respectively, 28 and 12 duplicates respectively)

Search repeated on 08.05.2020 for all search engines (3 months passed from initial search).

Pubmed

(cardiomyopathy OR cardiomyopathies OR hypertrophic cardiomyopathy OR hypertrophic obstructive cardiomyopathy OR HCM OR HOCM OR dilated cardiomyopathy OR DCM OR arrhythmogenic right ventricular dysplasia/cardiomyopathy OR arrhythmogenic right ventricular dysplasia OR arrhythmogenic right ventricular cardiomyopathy OR ARVD/C OR ARVD OR ARVC OR hypertrabeculation OR non-compaction OR non compaction OR LVNC) AND (speckle OR speckles OR strain OR STE OR STE-derived OR 2DSTE OR 2D-STE OR 2D STE OR deformation echocardiography OR deformation imaging OR speckle tracking) AND (child OR children OR pediatric OR paediatric OR adolescent OR adolescents OR newborn OR teen OR teens OR boy OR boys OR girl OR girls OR youth OR youths OR juvenile OR juveniles) AND (echo OR echography OR echocardiography OR echographic OR echocardiographic OR ultrasound OR ultrasonography OR sonography)

OVID

#1 (cardiomyopathy or cardiomyopathies or hypertrophic cardiomyopathy or hypertrophic obstructive cardiomyopathy or HCM or HOCM or dilated cardiomyopathy or DCM or arrhythmogenic right ventricular dysplasia*cardiomyopathy or arrhythmogenic right ventricular dysplasia or arrhythmogenic right ventricular cardiomyopathy or ARVD*C or ARVD or ARVC or hypertrabeculation or non-compaction or non compaction or LVNC).mp. [mp=title, abstract, original title, name of substance

word, subject heading word, floating sub-heading word, keyword heading word, organism supplementary concept word, protocol supplementary concept word, rare disease supplementary concept word, unique identifier, synonyms] OR (exp Cardiomyopathies/)

#2 (speckle or speckles or strain or STE or STE-derived or 2DSTE or 2D-STE or 2D STE or deformation echocardiography or deformation imaging or speckle tracking).mp. [mp=title, abstract, original title, name of substance word, subject heading word, floating sub-heading word, keyword heading word, organism supplementary concept word, protocol supplementary concept word, rare disease supplementary concept word, unique identifier, synonyms]

#3 (echo or echography or echocardiography or echographic or echocardiographic or ultrasound or ultrasonography or sonography).mp. [mp=title, abstract, original title, name of substance word, subject heading word, floating sub-heading word, keyword heading word, organism supplementary concept word, protocol supplementary concept word, rare disease supplementary concept word, unique identifier, synonyms] OR (exp Echocardiography/)

#4 (child or children or pediatric or paediatric or adolescent or adolescents or newborn or teen or teens or boy or boys or girl or girls or youth or youths or juvenile or juveniles).mp. [mp=title, abstract, original title, name of substance word, subject heading word, floating sub-heading word, keyword heading word, organism supplementary concept word, protocol supplementary concept word, rare disease supplementary concept word, unique identifier, synonyms]

Search: #1 AND #2 AND #3 AND #4

Scopus

TITLE-ABS-KEY((speckle OR speckles OR strain OR STE OR STE-derived OR 2DSTE OR 2D-STE OR "2D STE" OR "deformation echocardiography" OR "deformation imaging" OR "speckle tracking")) AND ((cardiomyopathy OR cardiomyopathies OR hypertrophic cardiomyopathy OR "hypertrophic obstructive cardiomyopathy" OR HCM OR HOCM OR "dilated cardiomyopathy" OR DCM OR "arrhythmogenic right ventricular dysplasia/cardiomyopathy" OR "arrhythmogenic right ventricular dysplasia" OR "arrhythmogenic right ventricular cardiomyopathy" OR {ARVD/C} OR

ARVD OR ARVC OR hypertrabeculation OR "non-compaction" OR "non compaction" OR LVNC)) AND ((echo OR echography OR echocardiography OR echographic OR echocardiographic OR ultrasound OR ultrasonography OR sonography)) AND ((child OR children OR pediatric OR paediatric OR adolescent OR adolescents OR newborn OR teen OR teens OR boy OR boys OR girl OR girls OR youth OR youths OR juvenile OR juveniles))

EMBASE

('cardiomyopathy' OR 'cardiomyopathies' OR 'hypertrophic cardiomyopathy' OR 'hypertrophic obstructive cardiomyopathy' OR 'HCM' OR 'HOCM' OR 'dilated cardiomyopathy' OR 'DCM' OR 'arrhythmogenic right ventricular dysplasia/cardiomyopathy' OR 'arrhythmogenic right ventricular dysplasia' OR 'arrhythmogenic right ventricular cardiomyopathy' OR 'ARVD/C' OR 'ARVD' OR 'ARVC' OR 'hypertrabeculation' OR 'non-compaction' OR 'non compaction' OR 'LVNC') AND ('speckle' OR 'speckles' OR 'strain' OR 'STE' OR 'STE-derived' OR '2DSTE' OR '2D-STE' OR '2D STE' OR 'deformation echocardiography‘ OR 'deformation imaging' OR 'speckle tracking') AND ('child' OR 'children' OR 'pediatric' OR 'paediatric' OR 'adolescent' OR 'adolescents' OR 'teen' OR 'teens' OR 'boy' OR 'boys' OR 'girl' OR 'girls' OR 'youth' OR 'youths' OR 'juvenile' OR 'juveniles') AND ('echo' OR 'echography' OR 'echocardiography' OR 'echographic' OR 'echocardiographic' OR 'ultrasound' OR 'ultrasonography' OR 'sonography')

Web of Science

#1 ALL=(cardiomyopathy OR cardiomyopathies OR hypertrophic cardiomyopathy OR hypertrophic obstructive cardiomyopathy OR HCM OR HOCM OR dilated cardiomyopathy OR DCM OR arrhythmogenic right ventricular dysplasia*cardiomyopathy OR arrhythmogenic right ventricular dysplasia OR arrhythmogenic right ventricular cardiomyopathy OR ARVD*C OR ARVD OR ARVC OR hypertrabeculation OR non-compaction OR non compaction OR LVNC)

#2 ALL=(speckle OR speckles OR strain OR STE OR STE-derived OR 2DSTE OR 2D-STE OR 2D STE OR deformation echocardiography OR deformation imaging OR speckle tracking)

#3 ALL=(child OR children OR pediatric OR paediatric OR adolescent OR adolescents OR newborn OR teen OR teens OR boy OR boys OR girl OR girls OR youth OR youths OR juvenile OR juveniles)

#4 ALL=(echo OR echography OR echocardiography OR echographic OR echocardiographic OR ultrasound OR ultrasonography OR sonography)

Search: #1 AND #2 AND #3 AND #4

EBSCO – CINAHL

((cardiomyopathy OR cardiomyopathies OR hypertrophic cardiomyopathy OR hypertrophic obstructive cardiomyopathy OR HCM OR HOCM OR dilated cardiomyopathy OR DCM OR arrhythmogenic right ventricular dysplasia/cardiomyopathy OR arrhythmogenic right ventricular dysplasia OR arrhythmogenic right ventricular cardiomyopathy OR ARVD/C OR ARVD OR ARVC OR hypertrabeculation OR non-compaction OR non compaction OR LVNC)) AND ((speckle OR speckles OR strain OR STE OR STE-derived OR 2DSTE OR 2D-STE OR 2D STE OR deformation echocardiography OR deformation imaging OR speckle tracking)) AND ((child OR children OR pediatric OR paediatric OR adolescent OR adolescents OR newborn OR teen OR teens OR boy OR boys OR girl OR girls OR youth OR youths OR juvenile OR juveniles)) AND ((echo OR echography OR echocardiography OR echographic OR echocardiographic OR ultrasound OR ultrasonography OR sonography))

CENTRAL

#1 cardiomyopathy OR cardiomyopathies OR hypertrophic cardiomyopathy OR hypertrophic obstructive cardiomyopathy OR HCM OR HOCM OR dilated cardiomyopathy OR DCM OR arrhythmogenic right ventricular dysplasia OR arrhythmogenic right ventricular dysplasia OR arrhythmogenic right ventricular cardiomyopathy OR ARVD OR ARVD OR ARVC OR hypertrabeculation OR non-compaction OR non compaction OR LVNC OR restrictive cardiomyopathy OR RCM

#2 speckle OR speckles OR strain OR STE OR STE-derived OR 2DSTE OR 2D-STE OR 2D STE OR deformation echocardiography

#3 child OR children OR pediatric OR paediatric OR adolescent OR adolescents OR newborn OR teen OR teens OR boy OR boys OR girl OR girls OR youth OR youths OR juvenile OR juveniles

#4 echo OR echography OR echocardiography OR echographic OR echocardiographic OR ultrasound OR ultrasonography OR sonography

Search: #1 AND #2 AND #3 AND #4

Additional methodological data

Description of all echocardiographic data extracted

STE derived measurements of interest were: global (apical 4, 2 and 3 chamber average) or apical four chamber (A4C) left ventricular (LV) longitudinal strain (grouped together as S_l), right ventricle (RV) longitudinal strain (free wall 3 segments or global 6 segments), LV basal (6 segments at the mitral valve short axis view), mid (papillary muscle short axis view) or apical (apical short axis view) circumferential strain (S_c), LV basal, mid or apical radial strain (S_r), strain derived LV rotational mechanics (basal and apical rotation, twist and torsion) and STE derived dyssynchrony parameters

We extracted the following conventional echocardiographic parameters: LV and RV chamber size measurements, LV ejection fraction (LVEF), LV fractional shortening (FS), myocardial performance index (MPI), isovolumic contraction time (IVCT), right ventricular fractional area change (RV FAC), mitral annular plane systolic excursion (MAPSE), tricuspid annular plane systolic excursion (TAPSE), left ventricle diastolic function (E, A, E/A, E', E/E'), isovolumic relaxation time (IVRT), TDI derived systolic function (LV and RV peak S' velocity).

Numerical data extraction and transformations

Medians and interquartile range were converted to means (SD).(1) When results were only presented as plots, numerical values were obtained using scale based coordinate measurements. Combined mean (SD) of two independent subgroups, mean differences and standardized mean differences (SMD) between ICC and controls were calculated using the Cochrane recommended formulas.(2) When exact p values were not provided (e.g. "p<0.05" or comparison made in subgroups), they were calculated using the means, SD and sample size, by calculating the t statistics.(3) For p values <0.0001, a random value (between 0.0001 and 0.00001) was used for plotting only, to avoid graphical overlaps.

Risk of bias assessment

Risk of bias was assessed independently by D.M.D. and C.W. using the Newcastle-Ottawa scale for case control studies.(4) In one study, by Pieles et al. (2020)(5), the patient and control groups are HCM

with and without specific genotypes, and was considered a case-control study for the purpose of the risk of bias analysis, but one single HCM cohort, for the purpose of analysis. This scale uses three item groups: selection of cases/controls, their comparability and ascertainment of exposure. Two limitations present in most of the included papers were inherently linked to the characteristics of imaging studies: firstly, most control groups are healthy outpatients, not selected from the general population, and secondly, it is difficult to truly blind evaluations when pathological echocardiographic images are present. Also, in most studies, while it is specified that quality checks were completed when performing STE analysis, the exact number of cases where images were not usable, and how it impacted the final figures, are not clearly stated. As such, the lower scores in the selection and exposure categories due to these issues should not be seen as study flaws, but as general limitations in the research field. Studies were also subjectively and semi-qualitatively graded into “low”, “medium” and “high” risk of bias, based on the Newcastle-Ottawa total score, and sub-category scores.

Table S1. Summary of cardiac function by non-speckle tracking measurements						
	Source	ICC mean (SD)	Controls mean (SD)	Mean difference	p value (reported)	SMD
Dilated cardiomyopathy (DCM)						
LVEF, %	Jin et al,(6)	26.5 (13.3)	58.6 (5.9)	-32.1	<0.001	-3.5
	Friedberg et al,(7)	34 (12)	66 (7)	-32	<0.0001	-3.1
	Labombarda et al,(8)	31.3 (20.4)	61.7 (6.3)	-30.4	<0.0001	-2.0
	Agha et al,(9)	39.33 (9.3)	69 (7)	-29.67	<0.0001	-3.6
	Maher et al,(10)	49.7 (5.5)	66.6 (5)	-16.9	<0.001	-3.2
	Sabatino et al,(11)	39 (12)	60 (4)	-21	<0.01	-2.4
Septal S’, cm/s	Jin et al,(6)	4.6 (2)	7.4 (1)	-2.8	0.005	-1.9
	Agha et al,(9)	4.5 (0.8)	8 (1.6)	-3.5	<0.0001	-2.9
	Maher et al,(10)	4.7 (0.9)	6.7 (1.4)	-2	<0.001	-1.7
E/A	Agha et al,(9)	1.9 (0.8)	1.8 (0.3)	0.1	0.31	0.2
	Maher et al,(10)	1.4 (0.3)	1.5 (0.1)	-0.1	NS	-0.4
	Sabatino et al,(11)	1.8 (0.8)	1.8 (0.5)	0	NS	0.0
E/e'	Agha et al,(9)	12.9 (6.8)	6.8 (1.2)	6.1	<0.0001	1.2
	Sabatino et al,(11)	10.7 (4.2)	6.2 (1.2)	4.5	<0.01	1.5
Hypertrophic cardiomyopathy (HCM)						
LVEF, %	Forsey et al,(12)	64.5 (5.9)	64.2 (6.4)	0.3	0.86	0.005

	Prinz et al,(13)	64 (10)	64 (7)	0	NS	0
	Pieles et al,(5)	67.4 (11.1)				
	Sabatino et al,(11)	68 (7)	60 (4)	8	<0.01	1.4
Septal S’, cm/s	Forsey et al,(12)	8.2 (1.2)	8.9 (1.2)	-0.7	0.07	-0.6
	Pieles et al,(5)	6.7 (1.8)				
E/A	Prinz et al,(13)	1.9 (0.7)	2 (0.5)	-0.1	NS	-0.2
	Pieles et al,(5)	1.8 (0.6)				
	Sabatino et al,(11)	1.8 (0.9)	1.8 (0.5)	0	NS	0.0
E/e'	Forsey et al,(12)	6.15 (1.5)	6.5 (1.5)	-0.35	NS	-0.2
	Pieles et al,(5)	10.15 (3.9)				
	Sabatino et al,(11)	11.5 (5.5)	6.2 (1.2)	5.3	<0.01	1.4
Left ventricular non-compaction (LVNC)						
LVEF, %	Ari et al,(14)	68.9 (3.9)	71 (3.9)	-2.1	0.63	-0.5
	Yubbu et al,(15)	58 (11.6)	66 (6.3)	-8	not reported	-0.9
	Sabatino et al,(16)	54 (10.2)	64.5 (5.1)	-10.5	<0.001	-1.5
Septal S’, cm/s	Ari et al,(14)	7.7 (1.5)	8.1 (0.9)	-0.4	not reported	-0.3
E/A	Ari et al,(14)	1.6 (0.3)	1.7 (0.2)	-0.1	0.3	-0.4
	Sabatino et al,(16)	1.8 (0.5)	2 (0.9)	-0.2	NS	-0.3
E/e'	Ari et al,(14)	7.4 (2.8)	6.67 (4.5)	0.75	not reported	0.2
	Yubbu et al,(15)	9.5 (3.6)	7.7 (2.3)	1.82	not reported	0.6
	Sabatino et al,(16)	8.6 (2.8)	8 (3)	0.6	NS	0.2

Arrhythmogenic cardiomyopathy (ACM)						
FAC, %	Pieles et al,(17)	41	45	-4	<0.05	-0.6
RV S’, cm/s	Pieles et al,(17)	12.6	13.4	-0.8	NS	-0.3
TAPSE, mm	Pieles et al,(17)	22	23	-1	NS	-0.4
FAC, fractional area change; ICC, inherited cardiomyopathy; LVEF, left ventricular ejection fraction; RV, right ventricle; SMD, standardised mean difference; S’, tissue Doppler peak systolic velocity; TAPSE, tricuspid annular plane systolic excursion;						

**Table S2.** Summary of cardiac function and mechanics by speckle tracking measurements						
	Source	ICC mean (SD)	Controls mean (SD)	Mean difference	p value (reported)	SMD
Dilated cardiomyopathy (DCM)						
LV A4C S_l, %	Maher et al,(10)	-15.6 (3)	-21.6 (3.2)	6	<0.001	1.9
	Sabatino et al,(11)	-13.3 (5.3)	-21.5 (2.1)	8.2	<0.01	2
LV GLS, %	Agha et al,(9)	-11 (5.6)	-22.6 (3.3)	11.6	<0.0001	2.5
Basal S_c, %	Jin et al,(6)	-9.5 (3.6)	-15.7 (3.8)	6.2	<0.001	1.7
Basal S_r, %	Jin et al,(6)	15.7 (7.6)	42.2 (12.7)	-26.5	<0.001	-2.4
Mid S_r, %	Friedberg et al,(7)	18.5 (12)	49.8 (6)	-31.3	<0.001	-3.1
Basal Rot, degrees	Jin et al,(6)	-1.8 (1.2)	-4.2 (2.1)	2.4	0.006	1.3
Apical Rot, degrees	Jin et al,(6)	-0.9 (3.1)	5.9 (4.1)	-6.8	0.001	-1.8
LV twist, degrees	Jin et al,(6)	0.3 (2.1)	10.9 (4.6)	-10.6	<0.001	-2.7
Max delay S_l, ms*	Labombarda et al,(8)	143 (69)	77 (11)	66	<0.0001	1.3
Max delay S_r, ms*	Labombarda et al,(8)	178 (148)	50 (20)	128	<0.0001	1.2

Hypertrophic cardiomyopathy (HCM)						
LV A4C S_l, %	Forsey et al,(12)	-21.3 (2.2)	-21.4 (2.7)	0.1	0.95	0.04
	Sabatino et al,(11)	-16.7 (3)	-21.5 (2.1)	4.8	<0.01	1.9
	Pieles et al,(5)	-15.8 (4.7)				
Basal S_c, %	Forsey et al,(12)	-22.2 (2.7)	-22.7 (2.8)	0.5	0.62	0.2
	Pieles et al,(5)	-17.5 (5.6)				
Mid S_c, %	Forsey et al,(12)	-23 (3.2)	-22.2 (3.3)	-0.8	0.47	-0.2
Apical S_c, %	Forsey et al,(12)	-24.6 (3.8)	-22.4 (2.5)	-2.2	0.04	-0.7
Basal S_r, %	Pieles et al,(5)	31.8 (16.4)				
Basal Rot, degrees	Forsey et al,(12)	-8.7 (3.7)	-5.8 (1.9)	-2.9	0.0015	-1.1
	Prinz et al,(13)	-8.7 (4.3)	-4.9 (2.5)	-3.8	<0.05	-1.1
Apical Rot, degrees	Forsey et al,(12)	11.7 (4.3)	5.3 (1.9)	6.4	<0.0001	2.2
	Prinz et al,(13)	12.6 (8.5)	11.2 (6.7)	1.4	0.6	0.2
LV twist, degrees	Forsey et al,(12)	13.9 (4.7)	8.8 (2.5)	11.4	0.0008	3.4

LV torsion, degrees/mm	Prinz et al,(13)	2.8 (1.6)	1.9 (1)	0.9	<0.05	0.7
Left ventricular non-compaction (LVNC)						
LV A4C S_l, %	Ari et al,(14)	-18 (2.8)	-23.8 (3.3)	5.8	<0.001	1.9
	Yubbu et al,(15)	-17.5 (4.7)	-20.8 (2)	3.3	not reported	0.9
	Sabatino et al,(16)	-15.3 (4.2)	-19.6 (8.9)	4.3	<0.001	0.6
LV GLS, %	Sabatino et al,(16)	-16.1 (3.8)	-22.1 (2.2)	6	<0.001	2.1
Basal S_c, %	Yubbu et al,(15)	-19.1 (4.8)	-24.6 (3.9)	5.5	not reported	1.3
	Sabatino et al,(16)	-18.1 (5.3)	-23.4 (4.6)	5.3	<0.001	1.1
Mid S_c, %	Ari et al,(14)	-24.6 (4)	-27.5 (3.3)	2.9	0.019	0.8
	Yubbu et al,(15)	-16.3 (4.3)	-23.6 (4.6)	7.3	not reported	1.6
	Sabatino et al,(16)	-17.4 (5.3)	-26.5 (3.9)	9.1	<0.001	2.1
Apical S_c, %	Yubbu et al,(15)	-19.2 (5.5)	-30.3 (6.9)	11.1	not reported	1.8
	Sabatino et al,(16)	-14.9 (5.1)	-28.9 (8.1)	14	<0.001	1.9
Basal S_r, %	Yubbu et al,(15)	34.3 (10.8)	39.8 (11.1)	-5.5	not reported	-0.5

	Sabatino et al,(16)	26 (10)	59.3 (5)	-33.3	<0.001	-4.7
Mid S_r, %	Yubbu et al,(15)	26.8 (9.2)	40.4 (13.4)	-13.6	not reported	-1.2
	Sabatino et al,(16)	26.5 (16)	50.6 (6)	-24.1	<0.001	-2.3
Apical S_r, %	Yubbu et al,(15)	23.5 (8.6)	44.2 (14.5)	-20.7	not reported	-1.7
	Sabatino et al,(16)	23.7 (15)	41.1 (8)	-17.4	<0.001	-1.6
Basal Rot, degrees	Nawayotu et al,(18)	-7.1 (3.1)	-7.4 (2.2)	0.3	not reported	0.1
	Sabatino et al,(16)	-3.7 (2.1)	-4.3 (2.1)	0.6	<0.001	0.3
Apical Rot, degrees	Nawayotu et al,(18)	5.9 (3.8)	8.1 (3.1)	-2.2	not reported	-0.6
	Sabatino et al,(16)	0.1 (3.7)	7.2 (4.9)	-7.1	<0.001	-1.6
LV twist, degrees	Nawayotu et al,(18)	10 (6.6)	15.1 (4.1)	-5.1	not reported	-0.9
	Sabatino et al,(16)	3.8 (3.8)	13.5 (5.7)	-9.7	<0.001	-1.9
LV torsion, degrees/mm	Nawayotu et al,(18)	1.7 (1.2)	2.5 (0.7)	-0.8	not reported	-0.8
Arrhythmogenic cardiomyopathy (ACM)						
RV GLS	Pieles et al,(17)	-21 (4)	-25 (3)	4	<0.001	1.1

RV free wall S_l	Pieles et al,(17)	-19 (5)	-24 (4)	5	<0.001	1.1
RV apical lateral S_l	Pieles et al,(17)	-17 (7)	-20 (7)	3	<0.01	0.4
RV apical septum S_l	Pieles et al,(17)	-20 (7)	-23 (7)	3	<0.01	0.4

A4C, apical 4 chamber view; GLS, global longitudinal strain; ICC, inherited cardiomyopathy; LV, left ventricle; Rot, rotation; RV, right ventricle; S_c, circumferential strain; S_l, longitudinal strain; S_r, radial strain; SMD, standardised mean difference.

*Labombarda et al.(8) reports additional dyssynchrony parameters, only two have been selected.

REFERENCES

1. Wan X, Wang W, Liu J, Tong T. Estimating the sample mean and standard deviation from the sample size, median, range and/or interquartile range. BMC Med Res Methodol. 2014;14(1):1–13.

2. Higgins J, Li T, Deeks J. Chapter 6: Choosing effect measures and computing estimates of effect. In: Higgins J, Thomas J, Chandler J, Cumpston M, Li T, Page M, et al., editors. Cochrane Handbook for Systematic Reviews of Interventions version 60 (updated July 2019). Cochrane; 2019.

3. Ludbrook J. Practical Statistics For medical Research. Vol. 61, Australian and New Zealand Journal of Surgery. Chapman and Hall; 1991. p. 963–4.

4. Wells GA, Tugwell P, O'Connell D, Welch V, Peterson J, Shea B, et al. The Newcastle-Ottawa Scale (NOS) for assessing the quality of nonrandomized studies in meta-analyses. 2015;

5. Pieles GE, Alkon J, Manlhiot C, Fan C-PS, Kinnear C, Benson LN, et al. Association between genetic variants in the HIF1A-VEGF pathway and left ventricular regional myocardial deformation in patients with hypertrophic cardiomyopathy. Pediatr Res. 2020 May 6;1–10.

6. Jin SM, Noh C Il, Bae EJ, Choi JY, Yun YS. Decreased Left Ventricular Torsion and Untwisting in Children with Dilated Cardiomyopathy. J Korean Med Sci. 2007;22(4):633.

7. Friedberg MK, Slorach C. Relation Between Left Ventricular Regional Radial Function and Radial Wall Motion Abnormalities Using Two-Dimensional Speckle Tracking in Children With Idiopathic Dilated Cardiomyopathy. Am J Cardiol. 2008 Aug 1;102(3):335–9.

8. Labombarda F, Blanc J, Pellissier A, Stos B, Gaillard C, Bajolle F, et al. Health-e-Child Project: Mechanical Dyssynchrony in Children with Dilated Cardiomyopathy. J Am Soc Echocardiogr. 2009 Nov;22(11):1289–95.

9. Agha HM, Ibrahim H, El Satar IA, El Rahman NA, El Aziz DA, Salah Z, et al. Forgotten

Right Ventricle in Pediatric Dilated Cardiomyopathy. Pediatr Cardiol. 2017 Apr 1;38(4):819–27.

10. Maher E, Elshehaby W, El Amrousy D, El Razaky O. Left Ventricular Layer-Specific Myocardial Strains in Children with Recovered Primary Dilated Cardiomyopathy: What Lies Beneath the Iceberg? Pediatr Cardiol. 2020 Jan 1;41(1):101–7.

11. Sabatino J, Di Salvo G, Prota C, Bucciarelli V, Josen M, Paredes J, et al. Left Atrial Strain to Identify Diastolic Dysfunction in Children with Cardiomyopathies. J Clin Med. 2019 Aug 17;8(8):1243.

12. Forsey J, Benson L, Rozenblyum E, Friedberg MK, Mertens L. Early changes in apical rotation in genotype positive children with hypertrophic cardiomyopathy mutations without hypertrophic changes on two-dimensional imaging. J Am Soc Echocardiogr. 2014 Feb 1;27(2):215–21.

13. Prinz C, Faber L, Horstkotte D, Körperich H, Moysich A, Haas N, et al. Evaluation of left ventricular torsion in children with hypertrophic cardiomyopathy. Cardiol Young. 2008 Apr 6;24(2):245–52.

14. Ari ME, Cetin II, Kocabas A, Ekici F, Ceylan O, Surucu M. Decreased Deformation in Asymptomatic Children with Isolated Left Ventricular Non-compaction and Normal Ejection Fraction. Pediatr Cardiol. 2016 Jan 1;37(1):201–7.

15. Yubbu P, Nawaytou HM, Calderon-Anyosa R, Banerjee A. Diagnostic value of myocardial deformation pattern in children with noncompaction cardiomyopathy. Int J Cardiovasc Imaging. 2018 Oct 1;34(10):1529–39.

16. Sabatino J, Di Salvo G, Krupickova S, Fraisse A, Prota C, Bucciarelli V, et al. Left Ventricular Twist Mechanics to Identify Left Ventricular Noncompaction in Childhood. Circ Cardiovasc Imaging. 2019 Apr 1;12(4):e007805.

17. Pieles GE, Grosse-Wortmann L, Hader M, Fatah M, Chungsomprasong P, Slorach C, et al.

Association of Echocardiographic Parameters of Right Ventricular Remodeling and Myocardial Performance With Modified Task Force Criteria in Adolescents With Arrhythmogenic Right Ventricular Cardiomyopathy. Circ Cardiovasc Imaging. 2019 Apr 1;12(4).

18. Nawaytou HM, Montero AE, Yubbu P, Calderón-Anyosa RJC, Sato T, O'Connor MJ, et al. A Preliminary Study of Left Ventricular Rotational Mechanics in Children with Noncompaction Cardiomyopathy: Do They Influence Ventricular Function? J Am Soc Echocardiogr. 2018 Aug 1;31(8):951–61.
